# Generic health literacy of adults in Germany. Results of the Panel ‘Health in Germany’ 2024

**DOI:** 10.25646/14016

**Published:** 2026-04-01

**Authors:** Susanne Jordan, Simon Löbl

**Affiliations:** Robert Koch Institute, Department of Epidemiology and Health Monitoring, Berlin, Germany

**Keywords:** Health literacy, Adults, Survey and questionnaire, Population, HLS_19_-Q12, Gender, Age, Education

## Abstract

**Background:**

Generic health literacy (HL) encompasses cross-thematic and cross-contextual skills, motivation and knowledge in dealing with health information. Low HL is associated with higher healthcare needs and unfavourable health behaviours.

**Method:**

Data from the RKI Panel 2024 (n = 26,817) will be used to provide a current overview of HL among adults. HL was measured using the HLS_19_-Q12. Weighted analyses were stratified by gender, age and education.

**Results:**

81.3 % of women and 81.0 % of men have lower HL. With increasing age, the proportion of women with lower HL tends to decrease up to the 65 – 79 age group, whereas this trend is not observed in men. The group with high education has the lowest proportion of lower HL in both sexes.

**Conclusions:**

The results indicate a substantial need for action promoting HL. This should not primarily focus on individual capabilities, but rather, services and structures should be designed in a way that enables health-literate behaviour.

## 1. Introduction

Health literacy encompasses the abilities, motivation and knowledge to access, understand, appraise and apply health information in a variety of life situations with the aim of making health-related decisions [1]. People acquire health literacy through everyday experiences and in social relationships, but also in organisations such as education and healthcare system [2]. The design of the healthcare system, the way health information is presented and evidence-based, especially on the internet, or the quality of doctor-patient communication impose different demands on individuals. Therefore, health literacy is understood as a relational concept [3]. Health literacy should consequently not be viewed primarily as an individual ability, but rather as describing the fit between contextual requirements and individual abilities.

Studies on health literacy in Germany show that a large portion of the population has relatively low health literacy [4–7]. This empirical observation is highly relevant to public health, as low health literacy is often associated with poorer health, unfavourable health behaviours, more hospitalizations and higher use of emergency services, but less frequent use of screening [3, 8–10]. Based on these observations, there are increasing efforts to promote health literacy in Germany [11].

In order to identify needs for promoting health literacy at the population level and to monitor developments in health literacy among the population, the concept of *generic health literacy* is used both nationally and internationally [3, 12, 13]. The measurement tools used for this purpose provide cross-thematic and cross-contextual information about respondents’ self-assessed difficulties in dealing with health information [14]. Frequently used measurement tools are the Health Literacy Questionnaire (HLQ) and the HLS_19_-Q12 questionnaire [15].

## 2. Methods

The assessment of generic health literacy of the population in Germany was based on data from the 2024 annual survey from the panel ‘Health in Germany’ of the Robert Koch Institute (RKI Panel 2024). The sample was drawn from a double-stratified random selection of individuals aged 18 and older in private households. Participation was possible online or written-postal. A detailed description of the methodology of the RKI Panel 2024 can be found elsewhere [16]. All available cases (available case analysis) and thus data from n = 26,817 respondents were used for the cross-sectional analyses.

The measurement of generic health literacy was based on the validated German short version of the ‘Health Literacy Survey 2019’ (HLS_19_-Q12) instrument. The HLS_19_ instrument used in this study was developed within the ‘HLS_19_ – The International Health Literacy Population Survey 2019 – 2021’ of the M-POHL network (‘WHO Action Network on Measuring Population and Organisational Health Literacy’) [6, 17]. It comprises 12 single items that individuals use to assess their difficulties in accessing, understanding, appraising and applying relevant health information on a four-point Likert scale from very easy to very difficult. The overall index (type P) was calculated by transforming the sum of the numerical values of the twelve items (1 = very difficult, 2 = difficult, 3 = easy, 4 = very easy) to a 0 – 100 scale, provided that at least 10 out of 12 valid responses were available for a person [6, 18]. The different health literacy levels were determined using the thresholds proposed by M-POHL for the overall index [6] and were given the neutral labels ‘low’, ‘rather low’ (summarised as ‘lower’), ‘rather high’ and ‘high’ health literacy (the labels differ from those used by M-POHL).

The analyses with 95 % confidence intervals (95 % CI) were performed for all available cases, stratified by gender identity (categories: women, men, other; the latter group is excluded from the analysis due to the small number of cases (n = 61)), age groups and education (according to Comparative Analysis of Social Mobility in Industrial Nations, CASMIN [19]). Sample points were taken into account for the calculation of robust 95 % confidence intervals. The calculations were weighted to correct for deviations of the sample from the population structure in terms of age, gender, federal state, municipality class size, education and household size [16]. Adjusted Wald tests were performed for all reported group comparisons. Differences were considered statistically significant if the p-value was less than 0.05.

**Key messages**

► Approximately four-fifths of women and men fell into the category of ‘lower generic health literacy’.
► The proportion of lower health literacy tended to decrease with age in women (except for those aged 80 and older). In men, no comparable decrease was observed.
► Significant differences were observed between the educational groups in both genders across the three middle age groups. Individuals in the high education group had the lowest proportion of lower health literacy.

## 3. Results

**Figure 1:**
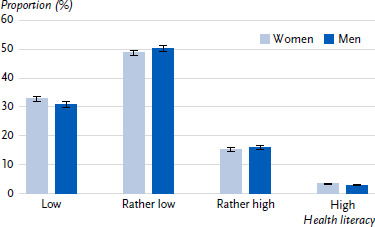
Distribution of generic health literacy in the adult population in Germany by gender (n = 14,674 women, n = 12,143 men). Source: RKI Panel 2024

The analyses showed that the largest group among both women and men had rather low health literacy (48.6 % among women; 50.1 % among men) (Figure 1). The smallest group was that with high health literacy, accounting for 3.4 % among women and 3.0 % among men. There were no significant differences between genders.

Analysis of the indicator ‘lower generic health literacy’ revealed that about four fifths of the surveyed women (81.3 %) and men (81.0 %) fell into this category (Table 1). The proportion of women with lower health literacy tended to decrease across the older age groups, with the exception of those aged 80 years and older. Among men, no comparable decrease across age groups was observed. Among them, a significant reduction in the proportion was only found between the 30 – 44 and the 45 – 64 age groups. The largest differences among women were observed between the youngest age group and those aged 65 – 79, and among men between 30 – 44 and those aged 80 and older. When examining age groups by gender, a significant difference was only found in the youngest age group, where the proportion with lower health literacy among women was 5.8 percentage points lower than among men. Significant differences between education groups were observed for both genders in the three middle age groups. The high education group consistently showed the lowest proportion of lower health literacy compared with the low and medium education groups.

## 4. Discussion

The results of the RKI Panel 2024 show that the majority of the population has lower generic health literacy. This means that people report difficulties in dealing with health information with regard to various demands in different life situations that affect personal health decisions and their health. A similarly high proportion has also been observed in other recent studies from Germany that assessed health literacy using the HLS_19_-Q12 ([4]: 75.8 %, [6]: 77 %). The educational differences identified in the study confirm the positive association between education and health literacy reported in previous research [3–5, 7]. Our study showed this for both women and men. The situation is different for age group differences, which are not observed consistently across all studies. However, a study conducted in 2024 using the same measurement instrument likewise found higher proportions of low health literacy among younger age groups [4].

When interpreting the results, it should be noted that health literacy was assessed using a self-assessment instrument, therefore, effects of social desirability cannot be ruled out [20]. The present results provide information on perceived difficulties, but not about the reasons for the reported difficulties, which may be individual and contextual in nature. Further research is needed in the form of qualitative study designs or supplementary performance-oriented measurement instruments that assess functional and cognitive abilities [14, 21]. A strength of the study is the large number of participants and its representativeness of the adult German-speaking resident population. This was achieved through recruitment via residents’ registration offices and study-specific weighting [16].

The study findings indicate a substantial need for action to promote health literacy. In this context, gender-specific differences related to age and education need to be considered. The education, social, and health systems, with their numerous organisations, play a central role in promoting health literacy, as they design services and structures that enable health literacy [22]. The World Health Organisation therefore recommends addressing less on individuals and instead focusing on social values, organisations and political regulations that strongly influence the health literacy of the population [23].

## Figures and Tables

**Table 1:** Proportion of adults in Germany with lower^*^ health literacy by gender, age and education (n = 14,674 women, n = 12,143 men). Source: RKI Panel 2024

	Women		Men	
	%	(95 % CI)	%	(95 % CI)
**Total**	**81.3**	**(80.5 – 82.2)**	**81.0**	**(80.0 – 81.9)**
**18 – 29 years**	**85.9**	**(83.7 – 87.9)**	**80.1**	**(77.2 – 82.7)**
Low education group	85.5	(76.8 – 91.3)	78.5	(68.4 – 86.0)
Medium education group	86.4	(84.0 – 88.4)	81.8	(78.5 – 84.6)
High education group	85.0	(81.6 – 87.8)	74.6	(69.0 – 79.5)
**30 – 44 years**	**83.0**	**(81.1 – 84.7)**	**83.7**	**(81.6 – 85.7)**
Low education group	89.0	(81.3 – 93.7)	86.5	(80.4 – 90.9)
Medium education group	84.4	(82.1 – 86.4)	85.0	(82.1 – 87.4)
High education group	77.2	(74.4 – 79.8)	79.2	(76.2 – 82.0)
**45 – 64 years**	**80.4**	**(78.9 – 81.9)**	**81.1**	**(79.6 – 82.6)**
Low education group	83.7	(79.5 – 87.2)	85.3	(82.1 – 88.1)
Medium education group	80.9	(79.2 – 82.5)	81.6	(79.3 – 83.6)
High education group	73.8	(70.8 – 76.7)	74.0	(71.1 – 76.7)
**65 – 79 years**	**77.6**	**(75.7 – 79.3)**	**79.1**	**(77.5 – 80.7)**
Low education group	81.0	(78.0 – 83.6)	80.0	(77.2 – 82.6)
Medium education group	75.5	(72.9 – 78.0)	81.9	(79.2 – 84.3)
High education group	69.0	(64.5 – 73.2)	73.7	(70.7 – 76.4)
**≥ 80 years**	**81.3**	**(77.8 – 84.3)**	**77.1**	**(73.8 – 80.1)**
Low education group	82.8	(78.3 – 86.6)	80.3	(75.9 – 84.1)
Medium education group	79.6	(74.1 – 84.1)	73.9	(66.1 – 80.4)
High education group	70.7	(62.3 – 77.8)	71.7	(65.9 – 76.9)

^*^ = Low and rather low health literacy combined
